# Association between the levels of polyunsaturated fatty acids and blood lipids in healthy individuals

**DOI:** 10.3892/etm.2012.724

**Published:** 2012-09-26

**Authors:** SI-PING WANG, YAN-HONG CHEN, HONG LI

**Affiliations:** Department of Special Service of the Chinese PLA General Hospital, Beijing 100853, P.R. China

**Keywords:** polyunsaturated fatty acid, ω-3, ω-6, ω-3 index, triglyceride

## Abstract

The levels of polyunsaturated fatty acids (PUFAs) in blood is closely associated with the status of the health of individuals; in particular, the lack of ω-3 fatty acids may lead to the development of numerous diseases. The aim of this study was to investigate the levels of PUFAs, and their correlation with triglycerides and other factors in blood. The levels of PUFAs and blood lipids were detected in 156 healthy individuals; the blood samples were tested by combined thin-layer and gas liquid chromatographic analysis. The level of ω-3 fatty acids was low in the subjects and the ω-3 index was 4.25%, while the ω-6:ω-3 ratio was at a satisfactory level. There was a strong inverse correlation between ω-3 fatty acids and triglycerides, and a strong positive correlation between ω-3 fatty acids and high density lipoprotein (HDL). Eicosapentaenoic acid and docosahexaenoic acid were inversely correlated with triglycerides, and positively correlated with HDL. In conclusion, ω-3 fatty acids are able to adjust the levels of blood lipids, and lower the levels of triglycerides, which may contribute to human health.

## Introduction

In recent years, polyunsaturated fatty acids (PUFAs) are an increasing concern for the health of individuals. PUFAs not only have a physiological function, but also play a key role in a variety of diseases, including cardiovascular disease, chronic inflammation, tumor and nervous system disease (1–3). PUFAs consist of the two main types of essential fatty acids in the human body, and play an important role in human health. Since PUFAs are acquired from food, individual dietary pattern and habit determine the PUFA levels in the human body and play a role in human health. For example, the three daily meals of Eskimos living in the Arctic Circle mainly depend on fish and meat. Eskimos are long-lived and the incidence rates of coronary heart disease, diabetes mellitus and other disease are extremely low. It is known that the substances affecting human health in this example are two types of fatty acids in food: eicosapentaenoic acid (EPA) and docosahexaenoic acid (DHA), or ω-3 PUFAs. PUFAs are essential fatty acids for the human body, and they are mainly divided into two classes: ω-3 PUFAs and ω-6 PUFAs. The former includes α-linolenic acid (ALA), EPA and DHA. The latter includes linoleic acid (LA), γ-linolenic acid (GLA) and arachidonic acid (AA). ω-3 PUFAs are mainly obtained from marine organisms, such as fish and crustaceans and are present in small amounts in edible vegetable oils. However, ω-6 PUFA is a main component of edible vegetable oils. Only specific ratios of ω-6 to ω-3 prevent disease incidence (4). A diet with a high ω-6 content and low ω-3 content is a dietary characteristic of modern society and it is one of the causes of coronary heart disease, diabetes mellitus and other diseases (5). At present there are no data on the PUFA intake of Chinese residents. Therefore, we randomly selected 156 healthy individuals receiving physical examinations to detect the levels of PUFAs to assess the ratio of ω-6 and ω-3. In addition, correlation analysis was carried out for PUFAs and other blood lipids.

## Materials and methods

### Subjects

A total of 253 individuals receiving physical examinations between January 2011 and May 2011 were randomly selected, including 165 males and 88 females. The average age was 48.33±9.79 years (range 27–77). The subjects assessed themselves as healthy and provided consent to the physical examination and detection of unsaturated fatty acids in blood. The patients fasted in the morning, and 2 ml blood was drawn, anticoagulated and sent for detection. In addition, body height and body weight were measured to calculate body mass index (BMI). This study was conducted in accordance with the Declaration of Helsinki. This study was conducted with approval from the Ethics Committee of the Chinese PLA General Hospital (Beijing, China). Written informed consent was obtained from all participants.

### Exclusion criteria

Patients with diabetes mellitus, coronary heart disease, various tumor-related diseases and patients receiving lipid-lowering drugs were excluded by comprehensive examination. The remaining healthy population included 156 cases.

### Detection methods

The international standard method was used for analysis of PUFAs (6), and capillary gas-liquid chromatography was used to detect PUFAs of the erythrocyte membrane. The main ω-3 PUFAs include EPA (20:5 ω-3), DHA (22:6 ω-3), ALA (18:3 ω-3). The main ω-6 PUFAs include LA (18:2 ω-6), AA (20:4 ω-6), docosatetraenoic acid (DTA, 20:3 ω-6) and docosapentaenoic acid (DPA, 22:5 ω-6). According to the peak area, the unsaturated fatty acid component was calculated. In addition, blood lipid levels of the sample, including triglyceride (TG), cholesterol (TC), high-density lipoprotein cholesterol (HDL-C) and low-density lipoprotein cholesterol (LDL-C), were detected. The ω-3 index and ω-6/ω-3 ratio were calculated.

### Statistical analysis

SPSS 13.0 software was used for statistical analysis. Detection results of unsaturated fatty acids and blood lipids are expressed as mean ± standard deviation, these were used to calculate ω-3 index and ω-6/ω-3 ratio. In addition, multiple linear regression analysis was used to assess the correlations of unsaturated fatty acid levels with gender, age and other blood lipids. P<0.05 was considered to indicate a statistically significant result.

## Results

### General data

We examined a total of 253 cases excluding patients with tumors, diabetes mellitus or coronary heart disease. The remaining 156 cases were healthy. Their general conditions such as age, TG, TC, HDL-C and LDL-C levels, BMI and blood pressure were measured, and the mean and standard deviation were obtained for each variable (Table I).

### PUFAs

Relative percentages of the main ω-6 and ω-3 fatty acids were evaluated in the healthy subjects. The maximum ω-3 index (percent of ω-3 in relation to other PUFAs) was 7.86% and its minimum value was 2.18%. The mean was 4.25±1.16%. The maximum ω-6/ω-3 ratio was 8.12 and its minimum value was 2.35. The mean was 4.63±1.12 (Table II).

### Correlations of PUFAs with blood lipids

Correlations of total ω-6, total ω-3 and their ratio with blood lipid, blood pressure and BMI were determined. Correlation coefficients for total ω-3 and ω-3 index with TG were −0.1576 and −0.1580, respectively (P<0.05), and correlation coefficients for total ω-3 and ω-3 index with HDL-C were 0.1585 and 0.1531, respectively (P<0.05). The correlation coefficient of ω-6 with age was -0.1501 (P<0.05). The relevant correlation coefficients and P-values are listed in Table III.

### Correlations of the main PUFA components with blood lipids

Main components of PUFAs include EPA, DHA and AA. Correlation coefficients of EPA and DHA with TG were −0.1742 and −0.1647, respectively (P<0.05), and correlation coefficients of EPA and DHA with HDL-C were 0.1332 and 0.1450, respectively (P<0.05). The correlation coefficient of AA with age was −0.1761 (P<0.05). The relevant correlation coefficients and P-values are listed in Table IV.

## Discussion

There are few data concerning the level of polyunsaturated fatty acids in the Chinese population, particularly in the healthy population. We analyzed PUFA levels in 253 healthy individuals receiving physical examinations at the Department of Special Service of the Chinese PLA General Hospital. After the patients with diabetes mellitus, tumors, coronary heart disease and other diseases were excluded, 156 healthy individuals were studied. The average age was 48.33±9.79 years. The average BMI was 25.9 and TG was 2.01, both of which were higher than normal levels, indicating that these individuals were overweight consistent with characteristics of modern society. The ω-3 index refers to the relative percentage of ω-3 in total PUFA levels, and it is often a key indicator of assessing the ω-3 level in the human body. The mean ω-3 index in our sample population was 4.25%. The internationally recommended ω-3 index is more than 8%; 4–8% represents the medium level, and less than 4% represents a deficiency of ω-3 (6). Therefore, ω-3 levels of our study population were nearly at the deficiency level, which is likely associated with the dietary structure in China where the majority of the diet depends on cereals while seafood consumption is relatively deficient. There are currently no data regarding the ω-3 intake in the dietary structure of Chinese residents, particularly for daily EPA and DHA intakes. The American Heart Association recommends that a patient with heart disease requires 1 g ω-3 daily, including EPA and DHA, and a healthy population without heart disease should consume fish twice weekly (approximately 0.5 g ω-3 daily). This significantly reduces the risk of heart disease (7–9). In China, there is no such reference guideline. According to the relevant data, ω-3 PUFAs prevent cardiovascular diseases, reduce inflammation and play an important role in reproductive and nervous system function (10–12), while ω-6 PUFAs mainly promote inflammatory reaction and certain functions are contrary to ω-3 PUFA functions (13,14). Therefore, only a suitable proportion of the two may improve health. In the present study, the ω-6/ω-3 ratio was 4.63. As the internationally recommended ω-6/ω-3 ratio ranges from 4:1 to 10:1, the ω-6/ω-3 ratio for our study population was at a relatively ideal level. The ω-6/ω-3 ratio is approximately 10:1 in Western countries and even reaches 50:1 in certain countries (15). Therefore, the ω-6/ω-3 ratio of our sample population was at a more optimum level than individuals in certain Western countries. Whether this is caused by the difference between Eastern and Western dietary structures requires further study.

In the present study, PUFA-related factors were further analyzed, particularly the correlations of PUFAs with other blood lipids. As shown in Table III, the total ω-3 level was negatively associated with TG level, and the correlation coefficient was −0.576, while the total ω-3 level was positively correlated with HDL-C, and the correlation coefficient was 0.1585. These are in agreement with certain studies (16,17). It was initially demonstrated that ω-3 reduces the risk of coronary heart disease and myocardial infarction. Therefore, it is considered a protective factor for coronary heart disease (15,17). This may be since ω-3 reduces serum TG levels and increases HDL-C levels. Our further analyses demonstrated that as two main components of ω-3, EPA and DHA are negatively associated with TG and positively associated with HDL-C, respectively, which is in agreement with other studies (16,18). Our results did not reveal a correlation of ω-3 with TC in the blood. The physiological effect of ω-6 remains unclear at present. ω-6 is mainly involved in inflammation in the body and is called the pathopoiesis factor (19). As a result, it was revealed that ω-6 is not correlated with TG or TC, while another study showed that ω-6 is negatively correlated with TG and TC (16). This may be due to the difference between Eastern and Western dietary habits or genetic differences. In addition, it is still believed that ω-6 is negatively correlated with age. Similarly, a main component of ω-6, AA, was found to also be negatively correlated with age (20,21), suggesting that human age is also an important factor influencing ω-6. No such phenomenon has been observed with ω-3.

The ratio of ω-6 and ω-3 is important for the human body (22,23). In modern society, particularly in Western countries, diet characteristics show high ω-6 content and low ω-3 content, which is contrary to the genetic metabolic gene background established since ancient times. In the last one hundred years, the ratio of ω-6 to ω-3 has become seriously unbalanced. In Western countries, it is usually in the range of 15:1 to 20:1, yet often higher. This type of unbalance is one of the reasons for modern diseases in modern society (24,25), including coronary heart disease, arthritis, cancer, osteoporosis, melancholia, xeroma and senile plaque (26,27). Therefore, it is necessary to carry out clinical intervention by increasing ω-3 intake and reducing ω-6 intake to maintain the balance of the two and thus prevent disease incidence. A ratio of ω-6/ω-3 of 4:1 is associated with the reduction in the mortality rate from cardiovascular diseases by 70% (27,28). The ratio of 2.5:1 inhibits proliferation of rectal cancer cells, while the ratio of 4:1 has no effect (29). A ratio ranging between 2:1 and 3:1 may inhibit rheumatic arthritis inflammation (30). In addition, the ratio of 5:1 benefits asthma, while a ratio of 10:1 is harmful for asthma (31,32). Therefore, the most appropriate ratio may vary with the disease. Our experimental results showed that the ratio of ω-6 to ω-3 of our population was relatively normal at 4.63 and remains satisfactory, complying with the internationally recommended ratio value ranging from 4:1 to 10:1.

Marinangeli *et al* (33) reported that oral administration of ω-3 could effectively reduce serious hypertriglyceridemia and reduced TG by 45%. The ω-3 dose included 465 mg/d EPA and 375 mg/d DHA, which further confirmed the effect of ω-3 from a clinical viewpoint. Dayspring (34) reported that for female population with high TG, the combination of oral administration of ω-3 and changes in dietary structure and life styles, including body weight loss, strengthening exercise and abstaining from smoking or drinking, had a marked efficiency. Therefore, oral ω-3 and a high cellulose diet are FDA-recognized treatment methods for serious hypertriglyceridemia. For the specific amount of oral ω-3, the FDA-recommended value ranging from 2 to 4 g/day may be used as a reference. Dietary therapy may avoid the adverse reaction caused by drug treatment and increase of blood lipid after drug administration termination. Therefore, it is feasible to treat hypertriglyceridemia by appropriately supplementing ω-3.

Although 156 cases were selected from 253 cases, they were relatively healthy. Yet, it is possible that individuals had unknown diseases or orally consumed certain unknown drugs, which may have affected the PUFA levels and influence the results. The present study was a cross-sectional study, and it remains unfeasible to completely clear their causal relationship. However, the ω-6/ω-3 ratio of our population was satisfactory and clearly lower than that reported by another study (35), which is likely in agreement with the dietary structure mainly composed of cereal foods in China.

We suggest that Chinese ω-3 fatty acid level is relatively low and is in a deficiency status according to our survey. Therefore, diet alone may not satisfy the requirements of the human body, and actively supplementing or increasing ω-3 in food is one of the important measures for enhancing or improving human health.

## Figures and Tables

**Table I:** General characteristics of the 156 healthy subjects.

Index	Minimum value	Maximum value	Mean	Standard deviation
Age (years)	27.00	77.00	48.33	9.79
TG (mmol/l)	0.50	19.92	2.01	2.30
TC (mmol/l)	0.99	7.20	4.85	0.95
HDL-C (mmol/l)	0.51	2.15	1.16	0.32
LDL-C (mmol/l)	0.53	5.71	2.89	0.82
BMI (kg/m^2^)	18.00	34.00	25.90	3.38
Systolic pressure (mmHg)	110.00	177.00	124.27	17.28
Diastolic pressure (mmHg)	53.00	112.00	78.67	10.16

TG, triglyceride; TC, cholesterol; HDL-C, high-density lipoprotein cholesterol; LDL-C, low-density lipoprotein cholesterol; BMI, body mass index.

**Table II:** Percentages of the main polyunsaturated fatty acids in the 156 healthy subjects.

Index	Minimum value	Maximum value	Mean	Standard deviation
LA (18:2 ω-6)	8.02	14.80	10.36	1.27
DTA (20:3 ω-6)	0.39	2.01	1.23	0.27
AA (20:4 ω-6)	9.34	18.02	14.77	1.52
DPA (22:5 ω-6)	0.09	1.01	0.44	0.17
Total ω-6	19.37	30.44	26.86	1.65
ALA (18:3 ω-6)	0.01	0.78	0.11	0.08
EPA (20:5 ω-6)	0.03	1.44	0.39	0.24
DHA (22:6 ω-6)	1.98	6.79	3.85	1.00
Total ω-3	3.59	9.77	6.08	1.26
ω-3 index	2.18	7.86	4.25	1.16
ω-6/ω-3 ratio	2.35	8.12	4.63	1.12

LA, linoleic acid; DTA, docosatetraenoic acid; AA, arachidonic acid; DPA, docosapentaenoic acid; ALA, α-linolenic acid; EPA, eicosapentaenoic acid; DHA, docosahexaenoic acid.

**Table III:** Correlations of polyunsaturated fatty acids with blood lipids [correlation coefficient (P-value)].

Item	TG	TC	HDL-C	LDL-C	BMI	Age
ω-3	−0.1576 (0.0251)^a^	0.0340 (0.3373)	0.1585 (0.0244)^a^	0.0882 (0.1367)	−0.0595 (0.02312)^a^	0.0917 (0.1281)
ω-6	0.0990 (0.1095)	−0.0062 (0.4694)	−0.0533 (0.2544)	−0.0768 (0.1702)	−0.0632 (0.2164)	−0.1501 (0.0307)^a^
ω-3 index	−0.1580 (0.0244)^a^	0.0473 (0.2787)	0.1531 (0.0282)^a^	0.1047 (0.0967)	−0.0553 (0.2465)	0.0779 (0.1669)
ω-6/ω-3 ratio	0.1909 (0.0085)^a^	0.0061 (0.4697)	−0.1131 (0.0800)	−0.0761 (0.1726)	−0.0404 (0.3082)	−0.1084 (0.0890)

a P<0.05. TG, triglyceride; TC, cholesterol; HDL-C, high-density lipoprotein cholesterol; LDL-C, low-density lipoprotein cholesterol; BMI, body mass index.

**Table IV:** Results of the correlations of EPA, DHA and AA with blood lipids [correlation coefficient (P-value)].

	TG	TC	HDL-C	LDL-C	BMI	Age
EPA	−0.1742 (0.0185)^a^	0.0851 (0.1453)	0.1332 (0.0487)^a^	0.0760 (0.1730)	0.0116 (0.4430)	0.0432 (0.2960)
DHA	−0.1647 (0.0199)^a^	0.0343 (0.3353)	0.1450 (0.0354)^a^	0.1028 (0.1009)	−0.0666 (0.2043)	0.0796 (0.1615)
AA	−0.0555 (0.2455)	−0.0218 (0.3936)	0.1210 (0.0662)	−0.0872 (0.1394)	−0.0719 (0.1861)	−0.1761 (0.0139)^a^
AA/EPA + DHA	0.1387 (0.0421)^a^	−0.0321 (0.3455)	−0.0938 (0.1222)	−0.1171 (0.0727)	−0.0402 (0.3091)	−0.1365 (0.0446)^a^

a P<0.05. EPA, eicosapentaenoic acid; DHA, docosahexaenoic acid; AA, arachidonic acid; TG, triglyceride; TC, cholesterol; HDL-C, high-density lipoprotein cholesterol; LDL-C, low-density lipoprotein cholesterol; BMI, body mass index.
